# P05.69. Removal of one or more arsenic related infections by using medicinal plants: findings from a rapid assessment study in Satkhira district of Bangladesh

**DOI:** 10.1186/1472-6882-12-S1-P429

**Published:** 2012-06-12

**Authors:** M Mollik

**Affiliations:** 1 Peoples Integrated Alliance Bangladesh, Bogra, Bangladesh

## Purpose

One of the more perplexing ground water problems currently facing Bangladesh is the high concentration of arsenic in drinking water, which poses a relatively large risk to human health of this region. Traditional health practitioners (THPs) of Bangladesh primarily use medicinal plants for treatment of various ailments. The selection of any medicinal plant is a closely guarded secret and is usually kept within the family. As a result, the use of medicinal plants varies widely between THPs of different areas within the country, and is based on both medicinal plant availability and the THP’s unique knowledge derived from practice. The aim of this present study was to conduct a survey amongst the THPs to learn more about the medicinal plants used to treat one or more arsenic related infections in the Satkhira district of Bangladesh. This area is unique in its proximity to the Sunderbans forest region and contains quite different medicinal plants compared to other parts of the country because of high salinity in the soil and water.

## Methods

Semi-structured questionnaires were administered to twenty-four traditional health practitioners to evaluate the THPs' perceptions and practice relating to causation and treatment of one or more arsenic related infections. The THPs described the signs, symptoms, and cause of one or more arsenic related infections. Details of the preparation and use of medicinal plants for management of one or more arsenic related infections were recorded.

## Results

In the present study, forty-one medicinal plant species belonging to thirty-nine genera and twenty-eight families were found to be used to treat one or more arsenic related infections in the Satkhira district.

## Conclusion

Information on indigenous use of medicinal plants has led to discovery of many medicines in use today. Scientific studies conducted on the medicinal plants may lead to discovery of more effective drugs than in use at present.

